# Detection of Hypoxanthine from Inosine and Unusual Hydrolysis of Immunosuppressive Drug Azathioprine through the Formation of a Diruthenium(III) System

**DOI:** 10.3390/bios11010019

**Published:** 2021-01-11

**Authors:** Marta Orts-Arroyo, Isabel Castro, José Martínez-Lillo

**Affiliations:** Instituto de Ciencia Molecular (ICMol), Universitat de València, c/ Catedrático José Beltrán 2, Paterna, 46980 València, Spain; marta.orts-arroyo@uv.es (M.O.-A.); isabel.castro@uv.es (I.C.)

**Keywords:** hypoxanthine, inosine, azathioprine, 6-mercaptopurine, biomarker, ruthenium

## Abstract

Hypoxanthine (hpx) is an important molecule for both biochemistry research and biomedical applications. It is involved in several biological processes associated to energy and purine metabolism and has been proposed as a biomarker for a variety of disease states. Consequently, the discovery and development of systems suitable for the detection of hypoxanthine is pretty appealing in this research field. Thus, we have obtained a stable diruthenium (III) compound in its dehydrated and hydrated forms with formula [{Ru(µ-Cl)(µ-hpx)}_2_Cl_4_] (**1a**) and [{Ru(µ-Cl)(µ-hpx)}_2_Cl_4_]·2H_2_O (**1b**), respectively. This purine-based diruthenium(III) system was prepared from two very different starting materials, namely, inosine and azathioprine, the latter being an immunosuppressive drug. Remarkably, it was observed that an unusual azathioprine hydrolysis occurs in the presence of ruthenium, thus generating hypoxanthine instead of the expected 6-mercaptopurine antimetabolite, so that the hpx molecule is linked to two ruthenium(III) ions. **1a** and **1b** were characterized through IR, SEM, powder and single-crystal X-ray Diffraction and Cyclic Voltammetry (CV). The electrochemical studies allowed us to detect the hpx molecule when coordinated to ruthenium in the reported compound. The grade of sensitivity, repeatability and stability reached by this diruthenium system make it potentially useful and could provide a first step to develop new sensor devices suitable to detect hypoxanthine.

## 1. Introduction

**Scheme 1:**
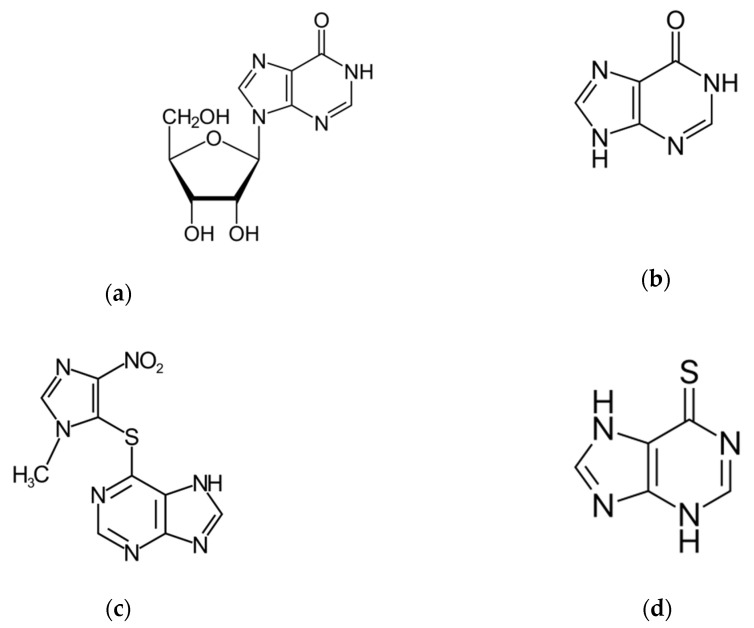
Molecular structure of: (**a**) Inosine, (**b**) Hypoxanthine, (**c**) Azathioprine and (**d**) 6-Mercaptopurine.

Hypoxanthine (6-hydroxypurine) is a deaminated form of adenine and a constituent of the nucleoside inosine (Scheme 1). It is formed during purine metabolism and is found in both tissues and body fluids of human beings and animals. The identification of hypoxanthine (hpx) as a biomarker for hypoxia has long been known [1,2]. More recently, hpx has also been proposed as a biomarker for colorectal cancer [3], Alzheimer’s disease [4], multiple sclerosis [5] and cardiac ischemia [6] and as checkpoint metabolite for other inflammatory processes [7] and toxicity levels [8]. In addition, hpx has been studied as a strong predictor of performance in highly trained athletes in sports and physical exertion, the hpx concentration indicating the training status and adaptation in consecutive phases of long training cycles [9,10]. Moreover, the determination of levels of hpx in meat and fish products has been established to be an important and convenient indicator of freshness and quality control in the food industry [11,12].

As a continuation of our interest in investigating biomolecule-based complexes of several metal ions [13,14,15,16,17] and their implementation in devices [18,19], we have studied the synthesis of ruthenium with some purine-based compounds that either contain hypoxanthine or could generate this analyte. Thus, we have investigated the products of the hydrolysis reactions with inosine and azathioprine (Scheme 1).

The nucleoside inosine is formed by a hpx molecule that is connected to a ribose ring through N(9)-glycosidic bond. It belongs to a group of purine antimetabolites recommended for treatment of measles and herpes infections, among others [20]. Studies on the inosine hydrolysis have been previously reported [21,22]. Azathioprine is a slow-release prodrug of the antimetabolite 6-mercaptopurine (Scheme 1), which is used as an anticancer and immunosuppressive drug [23,24]. It is used as an established clinical agent for the treatment of several pathologies, such as rheumatoid arthritis, ulcerative colitis, systemic lupus and Crohn’s disease, and also as an anti-rejection medication in human organ transplantation [23,24]. The N-methylnitroimidazolyl group of the azathioprine molecule protects the active form 6-mercaptopurine. Azathioprine undergoes hydrolysis in vivo to alter the metabolism and distribution of the drug toward its target [23,24].

Previously reported methods of determination of hpx include high-performance liquid chromatography [25,26,27,28], capillary electrophoresis [29,30], spectrophotometry [31,32] and electrochemiluminescence [33,34], which are usually costly and laborious and require a long analysis time. On the other hand, electrochemical methods offer several advantages, such as relatively simple and cheap instrumentation, high selectivity and sensitivity, high stability and rapid response time [35,36,37]. Nevertheless, given the wide range of potentially interfering purine-based compounds that can affect the detection of hpx generated in biological processes, more selective methods of analysis are needed.

**Figure 1:**
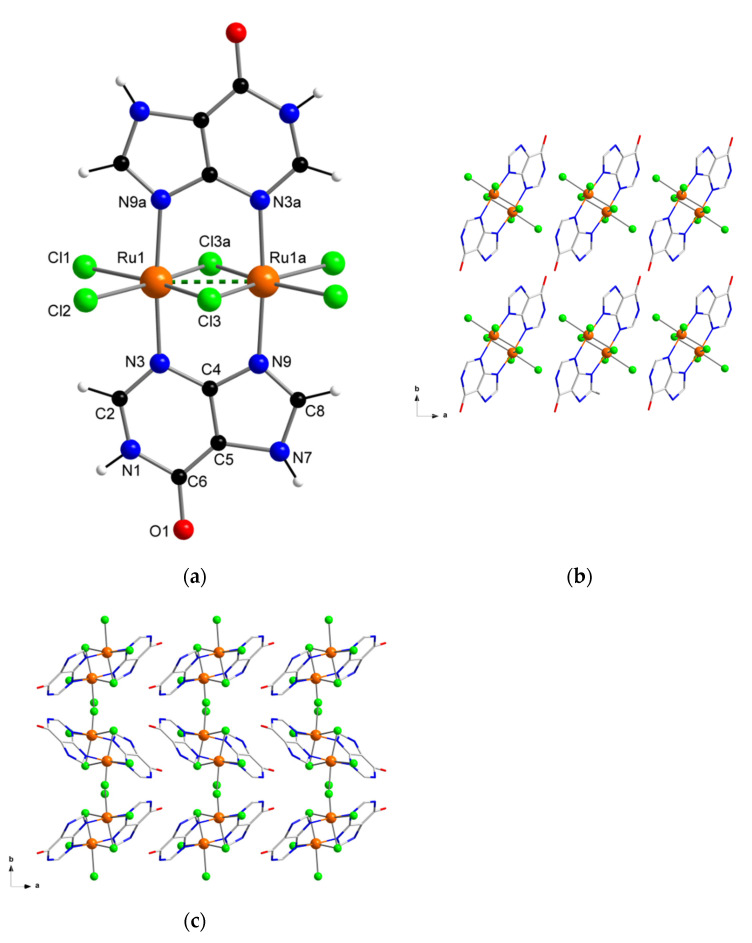
(**a**) Molecular structure of the [{Ru(µ-Cl)(µ-hpx)}_2_Cl_4_] complex in **1a** and **1b**; (**b**) view along the crystallographic *c* axis of a fragment of the crystal packing of **1a**; (**c**) view along the crystallographic *c* axis of a fragment of the crystal packing of **1b**.

Herein, we report the preparation, characterization and electrochemical properties of a new diruthenium system (Figure 1), which shows great stability and also selectivity for the hypoxanthine molecule, and therefore, could establish a first step to develop new sensor devices suitable for hypoxanthine detection.

## 2. Materials and Methods

### 2.1. Reagents and Instruments

**Figure 2:**
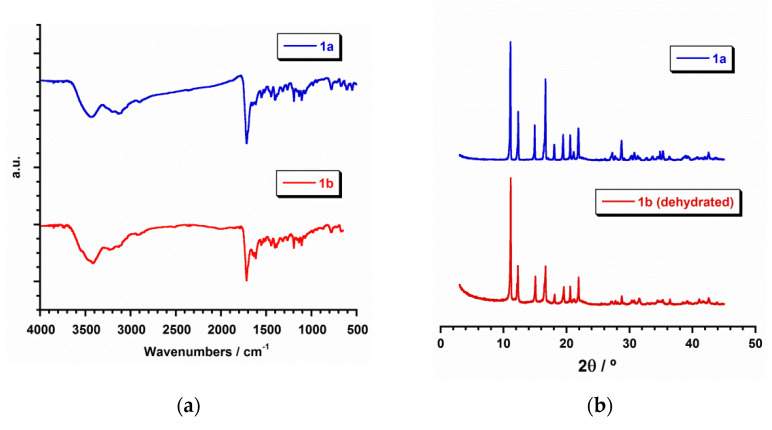
(**a**) IR spectra (Transmission mode) for **1a** (blue) and **1b** (red); (**b**) plot of the experimental powder X-ray diffraction (PXRD) patterns profile (2θ/°) in the range 0–50° for **1a** (blue) and dehydrated **1b** (red).

All of the manipulations were performed under aerobic conditions. Azathioprine and inosine were purchased from Alfa Aesar and Sigma Aldrich, respectively. Ruthenium precursors, RuCl_3_·H_2_O and K_2_[RuCl_5_(H_2_O)], and the rest of materials were used as-received and were of reagent grade. Elemental analyses (C, H, N) and X-ray microanalysis were performed by the Central Service for the Support to Experimental Research (SCSIE) at the University of Valencia. Scanning electron microscopy (SEM) images and results were obtained from a Hitachi S-4800 field emission scanning electron microscope. Infrared spectra (IR) of **1a** and **1b** (in the Supplementary Materials) were recorded with a PerkinElmer Spectrum 65 FT-IR spectrometer in the range of 400 to 4000 cm^−1^ (Figure 2).

Electrochemical studies were performed by using an Autolab/PGSTAT 204 scanning potentiostat operating at a scan rate range of 10–250 mV s^−1^. Cyclic voltammograms were carried out by using 0.1 M NBu_4_PF_6_ as supporting electrolyte and 0.001 M solutions of **1a**, inosine and azathioprine in dry dimethylformamide (dmf). The working electrode was a glassy carbon disk (0.32 cm^2^) that was polished with 1.0 μm of diamond powder, sonicated, washed with absolute ethanol and acetone and air dried. The reference electrode was AgCl/Ag, separated from the test solution by a salt bridge containing the solvent/supporting electrolyte, with platinum as an auxiliary electrode. All experiments were performed in standard electrochemical cells at 25 °C under argon. The investigated potential range was in the range of −2.0 to +2.0 V vs. AgCl/Ag. Ferrocene (F_c_) was added as internal standard at the end of all the measurements. The formal potentials were measured at a scan rate of 200 mV s^−1^ and were referred to the ferrocenium/ferrocene (F_c_^+^/F_c_) redox couple.

### 2.2. Preparation of the Compounds

#### 2.2.1. Synthesis of [{Ru(µ-Cl)(µ-hpx)}_2_Cl_4_] (**1a**)

A solvothermal reaction of K_2_[RuCl_5_(H_2_O)] (3.52 mg, 0.01 mmol) and inosine (2.66 mg, 0.01 mmol) was performed in HCl (4 mL, 3 M) at 90 °C for 3 days, followed by a 12-h cooling process to room temperature. Dark brown crystals of **1a** were obtained and were suitable for X-ray data collection. Yield: *ca*. 55%. Anal. Calcd. for C_10_H_8_Cl_6_N_8_O_2_Ru_2_ (**1a**): C, 17.5; H, 1.2; N, 16.3. Found: C, 17.7; H, 1.3; N, 16.5. IR peaks (KBr pellets, ν/cm^−1^): 3202(m), 3132(m), 3110(m), 3046(m), 2905(m), 1718(vs), 1653(m), 1555(m), 1522(w), 1447(m), 1407(m), 1318(m), 1267(m), 1196(s), 1160(w), 1137(m), 1118(s), 984(w), 779(m), 723(w), 674(m), 610(m), 550(m), 478(w) and 412(w).

#### 2.2.2. Synthesis of [{Ru(µ-Cl)(µ-hpx)}_2_Cl_4_]·2H_2_O (**1b**)

RuCl_3_·H_2_O (6.60 mg, 0.03 mmol) and azathioprine (6.80 mg, 0.03 mmol) reacted through a solvothermal reaction in HCl (4 mL, 3 M) at 90 °C for 3 days, followed by a 12-h cooling process to room temperature. Dark brown crystals of **1b** were thus obtained, which were suitable for X-ray data collection. Yield: *ca*. 72%. Anal. Calcd. for C_10_H_12_Cl_6_N_8_O_4_Ru_2_ (**1b**): C, 16.6; H, 1.7; N, 15.5. Found: C, 16.7; H, 1.9; N, 15.2. IR peaks (KBr pellets, ν/cm^−1^): 3433(br), 3202(m), 3133(m), 3110(m), 3047(m), 2905(m), 1718(vs), 1653(m), 1615(m), 1554(m), 1522(w), 1447(m), 1407(m), 1318(m), 1267(m), 1196(s), 1160(w), 1137(m), 1118(s), 984(w), 779(m), 723(w), 674(m), 610(m), 550(m), 478(w) and 412(w).

### 2.3. X-ray Data Collection and Structure Refinement

X-ray diffraction data from single crystals of dimensions 0.18 × 0.06 × 0.04 (**1a**) and 0.27 × 0.14 × 0.11 mm^3^ (**1b**) were collected on a Bruker D8 Venture diffractometer with graphite-monochromated Mo-K_α_ radiation (λ = 0.71073 Å). Crystal parameters and refinement results for **1a** and **1b** are summarized in Table 1. The structures were solved by standard direct methods and subsequently completed by Fourier recycling by using the SHELXTL software packages. The obtained models were refined with version 2017/1 of SHELXL against *F*^2^ on all data by full-matrix least squares [38]. In the two samples, all non-hydrogen atoms were anisotropically refined, whereas the hydrogen atoms of the hpx molecules were set in calculated positions and refined isotropically by using the riding model. The graphical manipulations were performed with the DIAMOND program [39]. The CCDC codes for **1a** and **1b** are 2046049 and 2046050, respectively. In addition, X-ray powder diffraction (PXRD) measurements were performed through a capillary sample holder in a PANalytical Empyrean diffractometer containing a hybrid monochromator (Cu-K_α1_ radiation) and a PIXcel detector.

## 3. Results and Discussion

### 3.1. Synthetic Procedure

By reacting K_2_[RuCl_5_(H_2_O)] with inosine (for **1a**) and RuCl_3_·H_2_O with azathioprine (for **1b**) in hydrochloric acid (3 M) solutions, we obtained the same diruthenium(III) complex, but in its dehydrated and hydrated forms [{Ru(µ-Cl)(µ-hpx)}_2_Cl_4_] (**1a**) and [{Ru(µ-Cl)(µ-hpx)}_2_Cl_4_]·2H_2_O (**1b**), respectively. In both cases, the synthesis process was performed by heating the reaction mixture at 90 °C through a solvothermal method, followed by a 12-h cooling process to room temperature as the crystallization technique. Thus, dark brown crystals of **1a** and **1b** were obtained in satisfactory yields. Inosine was chosen in this work as starting material because it is a suitable source of hypoxanthine (Scheme 1), and given the synthetic conditions, this purine nucleoside can easily undergo hydrolysis through a N(9)-glycosidic bond with a rapid release of hypoxanthine along with the ribose sugar [21]. The released hypoxanthine molecule acts as a ligand towards the ruthenium metal ions, generating the stable complex **1a**. In the case of the synthesis with azathioprine, **1b** was obtained as an unexpected product in a reaction which was intended to replace some of the Cl groups linked to the ruthenium(III) ions by the potentially chelating 6-mercaptopurine antimetabolite (Scheme 1). Instead, the species **1b** was formed. The hydrolytic reaction that azathioprine undergoes during its mechanism of drug action, that is, the cleavage of the N-methylnitroimidazolyl group from the sulfur atom, and therefore, the release of 6-mercaptopurine [23,24], in the presence of ruthenium(III) ions and in an acid medium, would be modified. After releasing the 6-mercaptopurine molecule, water attacks it, generating hypoxanthine, which in turn would lead to the formation of the species **1b**, so that the reported syntheses constitute new preparative methods to obtain purine-based Ru(III) compounds.

### 3.2. Description of the Crystal Structure

The crystal structure and exact chemical composition of **1a** and **1b** were established by single-crystal X-ray diffraction. **1a** and **1b** crystallize in the monoclinic system with space group *P*2_1_/*c* (Table 1). Their structures are made up of the neutral dinuclear [{Ru(µ-Cl)(µ-hpx)}_2_Cl_4_] units. Only in **1b** there are solvent molecules of crystallization, which are H_2_O molecules. In their asymmetric units, half a [{Ru(µ-Cl)(µ-hpx)}_2_Cl_4_] complex is in both **1a** and **1b**, and one H_2_O molecule of crystallization is also present in **1b** (Figure 1).

In the dinuclear complex of **1a** and **1b**, each six-coordinate Ru^III^ ion is bonded to four chloride ions and two nitrogen atoms (N3 and N9) from two hpx molecules in a distorted octahedral environment. No significant differences are found in the Ru–Cl [average value, 2.329(1) Å in **1a** and 2.310(1) Å in **1b**] and Ru–N [2.066(1) Å in **1a** and 2.061(1) Å in **1b**] bond lengths, which are similar in both compounds and are in agreement with those values found in previously reported Ru^III^ systems [40,41]. The two Ru^III^ ions are linked each other through two hpx molecules and a double Ru–Cl–Ru bridge and the short intramolecular Ru···Ru distance that is generated (ca. 2.6 Å) indicates the formation of a metal-metal bond (dashed line in Figure 1a). The hpx molecule in **1a** and **1b** is planar and its bond lengths and angles agree with those found in the literature for similar dinuclear complexes [42,43].

In the crystal lattice of **1a** and **1b** the dinuclear [{Ru(µ-Cl)(µ-hpx)}_2_Cl_4_] units pack in different ways (Figure 1b,c), because of their pseudopolymorphism. Thus, π···π stacking interactions between neighboring rings of coordinated hpx molecules occur in **1b** [with the shortest intercentroid distance being *ca*. 3.85 Å], whereas no π⋯π type interactions are observed in **1a**. Cl···π interactions between adjacent [{Ru(µ-Cl)(µ-hpx)}_2_Cl_4_] units take place in both Ru^III^ compounds [Cl···π distances varying in the ranges 3.16–3.37 and 3.46–3.73 Å for **1a** and **1b**, respectively]. In addition, H-bonding interactions contribute to stabilizing the crystal structure in both systems. Finally, the powder X-ray diffraction (PXRD) patterns of **1a** and **1b**, which are quite different to that of the hpx molecule [44], confirmed the homogeneity of their bulk samples (Figure 2).

### 3.3. Scanning Electron Microscopy (SEM)

**Figure 3:**
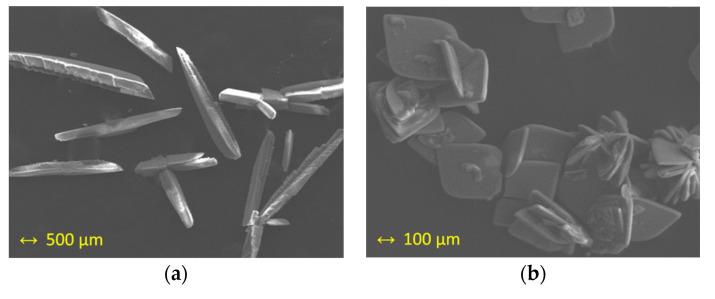
Scanning electron microscopy (SEM) images of: (**a**) crystals of **1a**; (**b**) crystals of **1b**.

The study of X-ray microanalysis through of scanning electron microscopy (SEM) gave an Ru/Cl molar ratio of 1:3 for both studied samples (**1a** and **1b**) and was performed as previously done for other ruthenium systems [45,46]. Sulfur was not detected in **1a** nor **1b**. The pseudopolymorphism of **1a** and **1b** was evident in the recorded images that are given in Figure 3, where crystals of **1a** are shown as needles, whereas crystals of **1b** are displayed as crystallized plates. 

### 3.4. Cyclic Voltammetry (CV)

**Figure 4:**
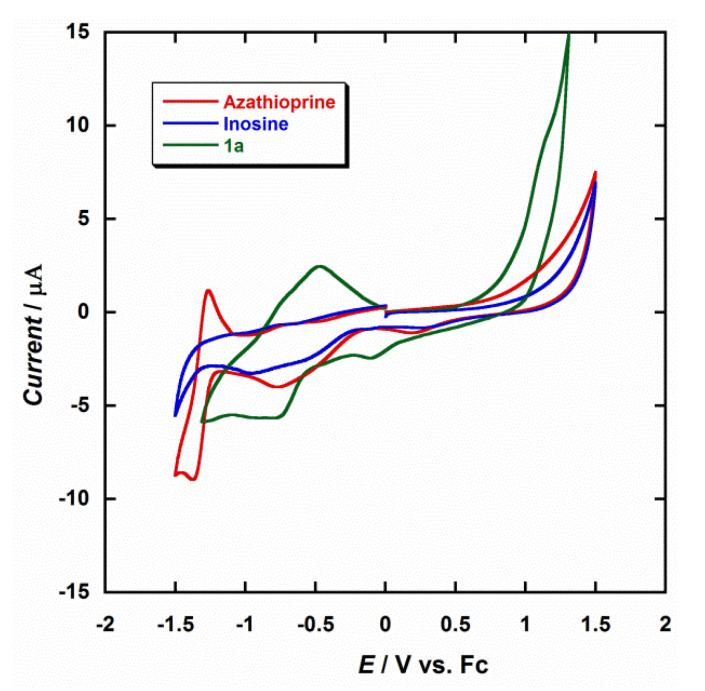
Cyclic voltammograms of **1a** (green), inosine (blue) and azathioprine (red) in dry dmf (0.1 M NBu_4_PF_6_) at 25 °C and scan rate 200 mV/s.

The electrochemical properties of the diruthenium(III) complex were investigated through cyclic voltammetry (CV) in dry dmf and at room temperature. Figure 4 shows a comparison of CV curves obtained in the same conditions for **1a**, inosine and azathioprine. The electrochemical behavior of azathioprine has been studied in detail in previous works [47,48,49,50]; nevertheless, we have included it for the purpose of our investigation. The most characteristic feature observed during the electrochemical reduction of azathioprine is the peak detected at a potential of about −1.30 V, which can be assigned to the reduction of the nitro group (-NO_2_) to the corresponding hydroxylamine (-NHOH) that the N-methylnitroimidazolyl moiety of the molecule contains [47,48,49,50]. The CV curve obtained for inosine is very similar to that of hypoxantine, which makes the detection of hypoxantine in the presence of inosine quite difficult [51,52].

Diruthenium complexes exhibiting metal–metal bonding have been extensively investigated for decades [53]. In general, this type of Ru_2_ compounds show electronic structures and redox properties that are unique [54,55,56]. Only one or up to three redox processes can be observed in the CV curves of these systems, depending on the solvent and the supporting electrolyte [54,55,56]. In dimethylformamide (dmf), only a single reduction would be expected, which would occur between 0.0 V and −0.30 V, so that the first reduction peak observed at −0.11 V for **1a** would be associated to the Ru^III^/Ru^II^ reduction process, which is very close to that reported for similar Ru_2_^III^ systems [54]. A second peak at −0.82 V would be generated by the influence of the analyte, in this case the hpx molecule (Figure 4). For testing repeatability of the CV curve of the studied Ru_2_^III^ system (**1a**), it was measured five times. Thus, a relative standard deviation of approximately 1.5% of the current response was obtained. It is evident from Figure 4 that there is a marked difference between the reported reductions waves for these three compounds, their current peaks being found to be well-resolved at the employed conditions, and the coordination of the hpx molecule being responsible of the CV curve observed for **1a**. Therefore, given the possibility of coexistence of hypoxanthine and inosine, or azathioprine, in singular biological processes or biomedical studies, our results can be used for the designing and development of new ruthenium-based devices that act as sensors for the detection of hypoxanthine. 

## 4. Conclusions

In summary, a new purine-based diruthenium(III) system of formula [{Ru(µ-Cl)(µ-hpx)}_2_Cl_4_] (hpx = hypoxanthine) has been prepared from two different starting materials, namely, inosine and azathioprine, the latter being an immunosuppressant drug. Remarkably, it was observed that during the synthetic process azathioprine undergoes hydrolytic reaction that in the presence of ruthenium generates hpx, instead of the expected 6-mercaptopurine antimetabolite, which in turn was detected through the formation of the diruthenium(III) system. The diruthenium(III) system was obtained in its dehydrated and hydrated forms and characterized by IR, SEM, powder and single-crystal X-ray diffraction and cyclic voltammetry (CV). The study on the CV curves allowed us to detect hpx when coordinated to ruthenium in the reported compound. The grade of sensitivity, repeatability and stability reached by this diruthenium system make it potentially useful and could provide a first step to develop sensor devices suitable to detect the hpx molecule. Further investigations on the synthesis and characterization of this type of ruthenium systems is now in progress for similar target molecules in our group.

## Figures and Tables

**Table 1:** Summary of the crystal data and structure refinement parameters for **1a** and **1b**.

Compound	1a	1b
CCDC	2046049	2046050
Formula	C_10_H_8_Cl_6_N_8_O_2_Ru_2_	C_10_H_12_Cl_6_N_8_O_4_Ru_2_
*M*_r_/g mol^−1^	687.08	723.12
Crystal system	monoclinic	monoclinic
Space group	*P*2_1_*/c*	*P*2_1_*/c*
*a*/Å	7.161(1)	8.714(1)
*b*/Å	10.720(1)	11.865(1)
*c*/Å	11.666(1)	10.286(1)
α/°	90	90
β/°	90.81(1)	112.32(1)
γ/°	90	90
*V*/Å^3^	895.52(9)	983.81(3)
*Z*	2	2
*D*_c_/g cm^−3^	2.548	2.441
μ(Mo-K_α_)/mm^−1^	2.611	2.390
*F*(000)	660.0	700.0
Goodness-of-fit on *F*^2^	1.170	1.066
*R*_1_ [*I* > 2σ(*I*)]	0.0726	0.0844
*wR*_2_ [*I* > 2σ(*I*)]	0.1811	0.2014

## Data Availability

Not applicable.

## Supplementary Materials

X-ray crystallographic data in CIF format for compounds **1a** and **1b** are available online at https://www.mdpi.com/2079-6374/11/1/19/s1.
